# Differing HIV vulnerability among female sex workers in a high HIV burden Indian state

**DOI:** 10.1371/journal.pone.0192130

**Published:** 2018-02-08

**Authors:** Megha Mamulwar, Sheela Godbole, Shilpa Bembalkar, Pranil Kamble, Nisha Dulhani, Rajesh Yadav, Chitra Kadu, Pradeep Kumar, Shivraj Lalikar, Shrikala Acharya, Raman Gangakhedkar, Arun Risbud, Srinivas Venkatesh

**Affiliations:** 1 National AIDS Research Institute [Indian Council of Medical Research], Pune, Maharashtra, India; 2 National Program Officer (Surveillance) National AIDS Control Organization, Strategic Information Management Unit [SIMU], Ministry of Health and Family Welfare, Government of India, New Delhi, India; 3 Joint Director STI, Monitoring &Evaluation, Maharashtra State AIDS Control Society, Mumbai, Maharashtra India; 4 Additional Project Director, Mumbai District AIDS Control Society, Mumbai, Maharashtra India; 5 Deputy Director General, National AIDS Control Organization, Ministry of Health and Family Welfare, Government of India, New Delhi, India; Massachusetts General Hospital, UNITED STATES

## Abstract

**Introduction:**

The HIV sentinel surveillance [HSS] conducted in 2010–11 among female sex workers [FSW] in the state of Maharashtra, India provided an opportunity to assess characteristics of different types of FSWs and their HIV risk. It is important for India’s National AIDS Control Program, to understand the differences in vulnerability among these FSW, in order to define more specific and effective risk reduction intervention strategies. Therefore, we analyzed data from HSS with the objective of understanding the HIV vulnerability among different types of FSW in Maharashtra.

**Material and methods:**

Cross sectional data collected as a part of HSS among FSWs in year 2010–11 from 21 sentinel sites in the state of Maharashtra were analyzed to understand the vulnerability and characteristics of different types of female sex workers based on their place of solicitation using multinomial logistic regression.

**Results:**

While the HIV prevalence was 6.6% among all FSWs, it was 9.9% among brothel based [BB], 9% among street based [SB] and 3.1% and 3.7% among home based [HB], and bar based [Bar-B] sex workers respectively. SB FSWs were least likely to be located in HIV low burden districts [ANC] [ARRR: 0.61[95% CI: 0.49, 0.77]], but were 6 times more likely to be recently [<1 year] involved in sex work [ARRR: 6.15 [95% CI: 3.15, 12.0]]. The number of clients of SB FSWs in the preceding week were lower than 11% [ARRR: 0.89 [95%CI: 0.87, 0.90]] as compared to the BB FSWs denoting lesser client load. The duration since last paid sex was shorter [ARRR: 0.94[95%CI: 0.91, 0.96]] as compared to the BB FSWs.

**Conclusion:**

Street based FSWs have emerged as one of the most vulnerable types of FSW with a high HIV prevalence similar to BB FSWs. Our study reveals that they have more frequent sex acts despite lower client loads, and are more likely to be located in districts highly affected by HIV (ANC prevalence >1%). We identify them as a group to be focused on for prevention interventions and it is likely that they would be easily amenable to novel interventions due to their higher literacy rate as compared to other typologies.

## Introduction

India ranks third in the world in terms of HIV disease burden[1]. In 2015, the National AIDS Control Organization [NACO] estimated that HIV prevalence in India was 0.26% among adults [15–49 yrs] with a burden of about 2.1 million persons living with HIV/ AIDS [PLHIV][1]. The Indian HIV epidemic is geographically diverse and is predominantly heterosexual. It is concentrated in high risk groups such as Female Sex Workers [FSW], Men having Sex with Men [MSM] and persons who inject drugs [PWID][2]. FSWs are considered as one of the key populations for prevention intervention largely due to the high turnover of sexual partners, number of sexual acts per day, higher HIV prevalence and the interactions with ‘bridging populations’. Bridging populations include individuals who have sexual partners in the highest risk groups as well as in the low risk population, usually spouses and thus form a transmission bridge between the HRG’s to the general population. Truckers and migrants are considered some of the bridging populations of focus by India’s prevention program. [3]

For over a decade, India’s National AIDS Control Program (NACP) has focused on prevention services through targeted interventions for key populations like FSW, MSM and PWID. Based on the findings of HIV surveillance, NACP has focused on achieving maximum coverage of high-risk populations with targeted interventions [TIs] in order to reduce HIV transmission[4]. These interventions are implemented by NACP funded non-governmental or community based organizations who follow specific guidelines for provision of prevention services. In 2011 these services included peer-based outreach and IEC [Information, Education and Communication] for HIV /AIDS awareness, condom negotiation skills, promotion and distribution of condoms and clinical services for prevention and management of STIs [3,5]

A community based survey among FSW, revealed an HIV prevalence of 2.2% at national level [6]. However HIV prevalence in India differs by states and even districts within states. Maharashtra, a high HIV burden state located on the western coast of India, has had a consistently high HIV prevalence among FSWs over the years [23.62% in 2005, 19.57% in 2006, 17.9 in 2007, 10.77% in 2008, 6.89% in 2012 and 7.4% in 2015 [7]. However there has been a decline in HIV prevalence, which is associated with increasing coverage of targeted interventions for FSWs and rising prevention intervention utilization by this population under NACP [4]. Despite the steady decline, HIV prevalence among FSWs is still significantly higher as compared to the general population and other Indian states [4]. Changes in characteristics and proportions of different types of sex workers in Maharashtra over time, could be one of the other likely reasons [4,8–10], however more extensive examinations are still warranted.

Traditionally, FSWs in Maharashtra used to be brothel based[11,12]. Recent mapping data from Maharashtra; however, highlights that a majority of FSWs now largely solicit in the non-brothel based settings rather than brothels [13]. FSWs in Maharashtra can be broadly grouped into two groups according to their place of solicitation. Those soliciting from brothels can be grouped as ‘Brothel Based’ [BB] and those soliciting from outside brothels as ‘Non Brothel Based’ [NBB][14]. NACP further categorizes NBB FSWs as ‘street based’ [SB], ‘home based’ [HB], ‘lodge/ dhaba’ based and ‘highway based’, depending on their place of solicitation [3]. The risk of acquiring HIV infection varies among different FSW typologies depending on their sexual behavior and use of condoms[15,16]. Street based sex workers are defined as those, who solicit clients on the street or in public places such as parks, railway/bus stations, markets, cinema halls. Home based or ‘secret’ sex workers usually operate from their homes, contacting their clients on the phone or through client referrals or through middlemen [e.g. auto drivers]. Lodge based sex workers are those, who work in a lodge [small hotels where rooms may be procured on hourly basis] and their clients are solicited by the manager/employee on the basis of profit sharing. Highway based sex workers are those, who recruit their clients from highways, usually from among long distance truck drivers[3].

It is also recognized, that there are certain occupations wherein a significant proportion engage in commercial sex fairly regularly, although their primary occupational identity may not indicate sex work. ‘Bar-girls’ who work in facilities like pubs and also engage in commercial sex work are classified as ‘Bar-based [Bar-B] sex workers. They are special to the state of Maharashtra and are common in the capital city of Mumbai[3].

The Behavioral Surveillance conducted in 2009 in the state of Maharashtra, revealed that NBB FSWs reported higher proportion of sex without condoms (vaginal or anal) as compared to their BB counterparts. However, they had fewer sexual partners in a week [17]. Their vulnerability to HIV infection was reported to be increased by acts of coercion and violence against them, resulting in less autonomy regarding condom use[17]. In addition, being less accessible to prevention programs increases their vulnerability[15,17]. A better understanding of the differing HIV vulnerability of FSWs may be useful to the program. Thus, prevention interventions for FSW’s need to be guided by the data on risks and vulnerabilities for HIV among them [18].

HIV Sentinel Surveillance (HSS) is a repeated cross sectional survey conducted at the same site, among the same population subgroups every 2 years which includes HIV testing along with collection of minimal demographic and risk related data [19]. The most recent round of HSS for which data are available, was conducted in 2010–11 among all high risk populations including FSWs in Maharashtra. While HIV prevalence data from this survey have been published in various reports. However specific details about different types of FSWs and their vulnerabilities have not been analyzed.

Hence in this study, we examined the data obtained from the HIV Sentinel Surveillance in Maharashtra conducted in 2010–11 to understand the vulnerability and characteristics of different types of female sex workers based on their place of solicitation. This was done in order to inform the National AIDS Control Program and help them in defining novel, more effective HIV risk reduction intervention strategies.

## Material and methods

### Ethics review and informed consent

The written informed consent and assent forms in the local language as well as Hindi and English were approved by the Ethics Committee of the Department of AIDS Control, Government of India [DAC GOI]. The present analysis was approved by Institutional Ethics Committee of National AIDS Research Institute [NARI].

### Study design

#### HIV Sentinel Surveillance (HSS)

HSS in India is a second generation HIV surveillance system devised to monitor the trends of HIV burden among various population groups and geographical areas[3]. HSS is conducted among Antenatal clinic attendees [ANC], groups at higher risk of HIV (Female Sex Workers [FSWs], Men having Sex with Men [MSMs], Injecting Drug Users [IDUs], Transgender [TGs]) and bridge populations of Single Male Migrants [SMMs] and Long Distance Truckers [LDTs].

Under HSS, the district level HIV prevalence among pregnant women is used as an indicator to categorize districts as high burden (>1% HIV prevalence among pregnant women attending antenatal clinic) or low burden (<1% HIV prevalence among pregnant women attending antenatal clinic). These categories are also used to guide programmatic interventions and funding.

The sentinel sites for the HIV surveillance among high risk groups are predetermined sites, identified by NACO and the regional institutes [19]. The Targeted Intervention [TI] project sites act as a sentinel surveillance sites for high risk population subgroups. Data and samples from 250 high risk individuals are collected over a 3 month period across each HSS site in the country during the surveillance round [3,19]. There were 21 such surveillance sites in Maharashtra state for FSWs in 2010. National AIDS Research Institute [NARI] was involved as a Regional Institute for surveillance and was responsible for site selection, training, monitoring implementation activities and data quality of surveillance. Standard technical guidelines were followed by all implementing sites nationwide described in detail elsewhere of which some are described below [19]. Selection of surveillance sites was based on epidemiologic need and availability of adequate numbers of the target population.

In this study we analyzed the data on FSWs collected from these sites. The inclusion criteria for FSWs in HSS 2010–11 was ‘Women between the ages 15 to 49 years who were engaged in consensual sex for money or payment in kind, as a means of livelihood in the last 6 months [19].

### Sample size and sampling

In HSS, a sample size of 250 individuals per high risk group site has been predetermined to provide a state level estimate of HIV prevalence among high risk groups. At each identified targeted intervention site, the sampling frame was the updated list of FSWs who were registered for prevention services and contactable in last 6 months, (also called as line-list). These line-lists were checked at NARI for inclusion criteria. After exclusion of ineligible cases and simple random sampling using SPSS software, 300 beneficiaries were randomly selected. The additional 50 numbers were selected to allow for replacements for refusals, inability to contact the individual or ineligibility. Trained peer educators working at the sentinel site were given the list of selected FSWs and they invited the selected FSWs to attend the drop in center to participate in the surveillance, using a standard message. If the selected FSW refused to participate or could not be contacted a replacement was provided until a sample of 250 was achieved [19].

In 2010–11, there were 21 sentinel sites in Maharashtra with an anticipated sample of 5250 FSWs[19]. Complete recruitment [250 i.e. 100%] was achieved by 15 (71%) sentinel sites, while the remaining 6 (29%) sites could recruit more than 90% of expected number of FSWs resulting in a final sample of 5179 FSWs.

Eligible respondents who provided a written informed consent/assent were included in the survey. In case, the respondent was in the age group of 15–17 years, informed assent was obtained from them, in addition to informed consent from the guardian/caregiver of the respondent [19]. A one page structured, 11 item questionnaire was completed after consent. Five drops of blood were collected on a specialized protein saver card [Whatman 903 protein saver card] through finger stick method. The cards were dried, packed and transported to the designated testing laboratory following standard operating procedures [20]. A two-test HIV testing protocol was used for testing the dried blood spots using standardized methods. Two ELISA tests, the first test of high sensitivity and the second of high specificity were used. The kits used were i] Microlisa HIV [J. Mitra & Co., New Delhi] and ii] Genedia HIV1/2 Elisa 3.0 [Green Cross Life Sciences Ltd, Korea]. Quality control of the testing was done at the National AIDS Research Institute, Pune for all positive samples and 2% of all negative samples [19].

The data form of each respondent was assigned a unique identification number along with date of sample collection at the sentinel site to follow the principle of unlinked anonymous testing [UAT]. This number was the only identifier used to identify the respondent’s blood sample. The respondents were offered prevention and care services at the site which included referral for HIV testing along with pre and post- test counseling.

### Study tool

A one page structured questionnaire was used to collect the following information: 1) age, 2) education, 3) reason for coming to the service point 4) current place of residence [rural/ urban] 5) duration of stay at current place of residence 6) type of sex work involved in [multiple options were allowed as brothel based, street based, home based, lodge based, dhaba based and others] 7) the duration for which she has been involved in sex work 8) duration since last paid sex 9) number of clients in the preceding week 10) other source of income, apart from sex work 11) use of injectable drugs without prescription for recreation, in the last 12 months.

### Data entry and data quality

Double data entry was done at NARI using an online data entry application nested within the Strategic Information Management System [SIMS], a web-based integrated monitoring and evaluation system developed by Department of AIDS Control [DAC], Government of India. This application included built-in ‘data matching’ and ‘data monitoring’ functions, ‘validation checks’ and customized report generation which were used for data cleaning and assuring quality. Cleaned and finalized data was provided to the Department of AIDS Control [DAC] for reporting and HIV estimations. The same dataset was used in this study.

### Statistical methods

We hypothesized that the vulnerability to HIV varies among different types (typology) of sex workers. Therefore, we compared the characteristics of brothel based (BB) FSWs with those of home, street and bar based FSWs. Since the number of lodge and dhaba-based FSWs was too small [n = 47] to consider as an independent typology, they were excluded from analysis. Additionally, 116 FSWs who reported more than one place of solicitation were also excluded from all stages of analysis to avoid the confounding effect of multiple typologies. Thus, after excluding these two subsets (116 FSWs reporting multiple typology and 47 lodge/dhaba based FSWs) from 5179, we analyzed the final data of 5016 FSWs in this study.

Overall HIV prevalence for the state of Maharashtra in 2010–11 was 0.42% among pregnant women attending ANC clinics[7]. However, at district level there were many districts with more than 1% prevalence among this low risk population. We created a variable of ‘TI district-categorization’ for our analysis. These districts were the ‘district of location’ for the targeted intervention (TI) sites from where FSWs were recruited for HSS. They were categorized into (a) ‘low burden district’ with <1% and (b) ‘high burden district’ with >1% HIV prevalence, among the pregnant women attending antenatal care clinics [ANC] in the same round of surveillance (2010–11] [7].

Characteristics of FSWs such as age, education, duration of stay, duration of sex work, number of clients, HIV status, and TI-district categorization were analyzed in detail as per sex work typology [Table 1]. These characteristics were compared using chi square test for discrete variables and Kruskal Wallis test for continuous variables.

**Table 1:** Characteristics of female sex workers by typology: Data from HIV Sentinel Surveillance, Maharashtra [2010–11].

		Brothel Based [BB]	Home Based [HB]	Bar Based [Bar-B]	Street Based [SB]	Overall	P value
		N = 1927	N = 1349	N = 1099	N = 641	5016	
**Mean Age**	In years [SD*]	29 [5.5]	32 [5]	27 [5.3]	32 [5]	30 [5.8]	<0.001
**Education** **n [%]**	Illiterate	1449 [75.4]	866 [64.7]	652 [59.4]	343 [53.8]	3310 [66.3]	<0.001
	Literate	474 [24.6]	472 [35.3]	445 [40.6]	295 [46.2]	1686[33.7]	
**Mean Duration of stay at current place of residence**	in years [SD]	8 [7.03]	15 [9.52]	11 [10.08]	12 [10.15]	10.8 [9.4]	<0.001
Mean Duration since LPS*	In days [SD]	3 [11.9]	7 [10.1]	9 [14.8]	3 [4.5]	5.3 [11.8]	<0.001
**Number of clients in preceding week**	Mean [SD]	23 [19]	4 [5]	2 [2]	7 [6]	11.2 [15]	<0.001
**Duration of sex work n[%]**	< 1 Year	50 [2.6]	16 [1.2]	73 [6.7]	22 [3.5]	161 [3.3]	<0.001
	1–3 Years	367 [19.1]	393 [29.3]	243 [22.2]	198 [31.5]	1201 [24.1]	
	3–5 Years	537 [28]	321 [23.9]	203 [18.5]	257 [40.9]	1318 [26.4]	
	> 5 Years	967 [50.3]	612 [45.6]	577 [52.6]	152 [24.2]	2308 [46.3]	
OSI apart from sex work* n[%]	Yes	6 [0.3]	410 [28.9]	324 [29.5]	93 [14.5]	833 [16.3]	<0.001
	No	1961 [99.7]	1009 [71.1]	774 [70.5]	548 [85.5]	4292 [83.6]	
**HIV Prevalence in FSWs n[%]**	Positive	191 [9.9]	42 [3.1]	41 [3.7]	58 [9]	332 [6.6]	<0.001
	Negative	1736 [90.1]	1307 [96.9]	1058 [96.3]	583 [91]	4684 [93.4]	
**TI-District categorisation^#^ n[%]**	<1%	1357 [70.4]	814 [60.3]	499 [45.4]	281 [43.8]	2951 [58.8]	<0.001
	≥1%	570 [29.6]	535 [39.7]	600 [54.6]	360 [56.2]	2065 [41.2]	

*SD- Standard deviation, LPS–Last paid sex, OSI- Other sources of inome

^#^TI-District categorisation—District with HIV prevalence [in pregnant women attending antenatal care clinic (ANC)] of <1% were considered as low HIV burden district and those with >1% HIV prevalence were considered as high HIV burden districts.

Multivariate multinomial regression was used to further understand the differences between female sex workers who were in brothel based sex work and those who were not [Table 2]. Typology (typology: Brothel based, Home based, Bar based and Street based FSW) was considered as a dependent variable while age, education, duration of stay at current place, duration since last paid sex [LPS], number of clients in preceding week, duration of sex work and TI-district categorization as covariates in this analysis. Brothel based category was considered as reference. Adjusted relative risk ratios [ARRR] along with 95% confidence intervals are reported.

**Table 2:** Comparison of characteristics of FSWs based in brothel with those not based in brothel using multinomial regression.

		Home Based		Bar Based		Street Based	
Characteristics		ARRR *and 95% CI	P value	ARRR and 95% CI	P value	ARRR and 95% CI	P value
**Age**	[In years]	1.03 [1.01, 1.05]	0.003	0.85 [0.83, 0.87]	<0.001	1.02 [1.00, 1.05]	0.027
**Education**	Literate	1.47 [1.20, 1.81]	<0.001	1.88 [1.48, 2.38]	<0.001	1.76 [1.41, 2.19]	<0.001
	Illiterate	1		1		1	
**Duration of stay at current place of residence**	[In years]	1.09 [1.07, 1.10]	<0.001	1.04 [1.03, 1.06]	<0.001	1.07 [1.05, 1.08]	<0.001
Duration since LPS**	[In days]	1.00 [0.99, 1.01]	0.974	0.99 [0.97, 0.98]	0.008	0.94 [0.91, 0.96]	<0.001
**Number of clients in preceding week**		0.82 [0.80, 0.83]	<0.001	0.59 [0.57, 0.62]	<0.001	0.89 [0.87, 0.90]	<0.001
**Duration of sex work**	< 1 Year	1.83 [0.85, 3.91]	0.121	3.65 [1.82, 7.30]	<0.001	6.15 [3.15, 12.0]	<0.001
	1–3 Years	3.23 [2.48, 4.20]	<0.001	0.74 [0.55, 1.01]	0.055	4.56 [3.35, 6.20]	<0.001
	3–5 Years	1.42 [1.12, 1.80]	0.004	0.50 [0.38, 0.67]	<0.001	3.84 [2.92, 5.06]	<0.001
	>5 Years	1		1		1	
**HIV**	Positive	0.23 [0.16, 0.34]	<0.001	0.28 [0.18, 0.44]	<0.001	0.69 [0.49, 0.98]	0.038
	Negative	1		1		1	
**TI-District categorisation^#^**	<1%	1.27 [1.04, 1.55]	0.022	0.46 [0.36, 0.58]	<0.001	0.61 [0.49, 0.77]	<0.001
	≥1%	1		1		1	

*ARRR- Adjusted Relative risk ratio

**LPS–Last paid sex

^#^TI-District categorisation—District with HIV prevalence [in pregnant women attending antenatal care clinic (ANC)] of <1% were considered as low HIV burden district and those with >1% HIV prevalence were considered as high HIV burden districts

## Results

Of the 5016 FSWs included in analysis, 1927 [38.4%] worked from brothels [BB], while 26.9%, 12.8% and 21.9% worked from homes(HB), bars (Bar B) and streets (SB) respectively. The overall HIV prevalence among FSWs was 6.6% and it was 9.9% among Brothel Based (BB), 3.1% in Home- based (HB), 3.7% in Bar-based (Bar-B) and 9% in Street-based (SB) FSWs.

### Background characteristics of FSWs

Overall, Bar-B FSWs were significantly younger with a mean age of 27 years (SD = 5.3) as compared to other typologies [mean age for HB FSWs: 32yrs (SD = 5), brothel Based FSWs: 29 yrs (SD = 5.5), SB FSWs: 32 yrs (SD = 5), p value< 0.001]. Although the proportion of illiterates was high overall, the bar based, home based and street based FSWs were more likely to be literate than BB FSWs [24.6%, p value < 0.001]. The BB FSWs mostly stayed at the place of residence for less than 10 years. As compared to BB FSWs [0.3%], other FSWs were more likely to have other sources of income apart from sex work [Table 1].

BB FSWs had the highest number of clients [mean = 23, SD = 19] in comparison with all other types of FSWs. Both the BB and SB FSWs were more likely to be sexually active in the last five days, with a mean duration of 3 days since last paid sex as compared to the other types [P value< 0.001]. Majority of SB FSWs were in sex work for 3–5 yrs [40.9%, P value< 0.001] and located in the high burden districts (HIV prevalence >1% in ANC) [56.2%, P value< 0.001].

### Comparative characteristics of different types of sex workers

Table 2 describes the comparative features of the HB, Bar B and SB FSWs with BB FSWs. On multinomial logistic regression it was observed that, with reference to BB FSWs, HB FSWs were 1.27 times more likely to be located in the low HIV burden districts [95% CI: 1.04,1.55]. The chance of HB FSWs being more recently [1–3 years] involved in sex work was 3.23 [95% CI: 2.48, 4.20] times higher than BB FSWs. HB FSWs were less likely to be HIV infected [ARRR: 0.23 [95% CI: 0.16, 0.0.34]. Additionally these FSWs were 1.03 times more likely to be older [95% CI: 1.01, 1.05], 1.47 times more likely to be literate [95% CI: 1.20, 1.81] and stably located at current place of residence [ARRR: 1.09 [95% CI: 1.07, 1.10]] [Table 2]. HB FSWs had lower client loads as compared to BB FSWs [ARRR: 0.82 (95%CI: 0.80, 0.83)].

Similarly, Bar-B FSWs were younger [ARRR: 0.85 [95% CI: 0.83, 0.87]] and more literate [ARRR: 1.88[95% CI: 1.48, 2.38]] than BB FSWs. Bar-B FSWs were 3.65 times more likely to be very recently involved in sex work [<1 years] [95% CI: 1.82, 7.30]. They were 0.46 times less likely to be located in the low HIV burden districts [ARRR: 0.46[95% CI: 0.36, 0.58]] as compared to BB FSWs. We found that Bar-B FSWs had a 41% decrease in ‘number of clients in the preceding week’ [ARRR: 0.59 [95%CI: 0.57, 0.62]], denoting a lower client load. They also showed significantly lower HIV prevalence [ARRR: 0.28 [95% CI: 0.18, 0.44]] as compared to BB FSWs.

HIV prevalence in SB FSWs was little lower than BB FSWs [ARRR: 0.69 [95%CI: 0.49, 0.98]] and they were least likely to be located in low HIV burden districts [ARRR: 0.61[95% CI: 0.49, 0.77]]. Street based FSWs were 6 times more likely to be recently involved in sex work [<1 year] [ARRR: 6.15 [95% CI: 3.15, 12.0]] as compared to BB FSWs. However SB FSWs were older [ARRR: 1.02 [95% CI: 1.00, 1.05]], more literate [ARRR: 1.76[95% CI: 1.41, 2.19]] and more stably located [ARRR: 1.07 [95% CI: 1.05, 1.08]] at current place of residence. The client load in the preceding week was11% [ARRR: 0.89 [95%CI: 0.87, 0.90]] lower however duration since last paid sex was significantly less than the BB FSWs [ARRR: 0.94[95%CI: 0.91, 0.96]].

## Discussion

Our analysis of HIV sentinel surveillance data, which included over 5000 FSWs from Maharashtra state, has revealed that SB FSWs (HIV prevalence 9%) in Maharashtra were as highly affected by the HIV epidemic as BB FSWs (HIV prevalence 9.9%). HIV prevalence among SB FSWs although lower than in BB FSWs, was higher than the national average (2.67%) for FSWs in India[20]. The vulnerability of the street based FSWs may be explained by the fact that, for the majority, sex work was the only source of income [85%] and they had significantly higher client load [>5 clients in preceding week] as compared to other FSWs. This is probably reflective of their monetary needs. In this respect, they were very similar to BB sex workers in our study.

Other studies have discussed that SB FSWs were less likely to use condoms with their clients, thus increasing their vulnerability to HIV infection [21]. Additionally due to their mobility and anonymous nature of solicitation, they may have less access to prevention interventions [15]. However HSS captured limited data on behavioral characteristics of FSWs, hence we could not assess the contribution of mobility of SB-FSWs or condom use in our study.

Our study also revealed that SB FSWs were more likely to be located in the high HIV burden districts (with HIV prevalence of >1% among ANC), suggesting that they may have a significant impact on the overall HIV transmission in the general population. Higher HIV prevalence among SB FSWs, high chances of being located in a high HIV burden district, higher literacy rate and similar vulnerabilities to BB FSWs making SB sex workers an important population, are some of the key leads from our study. Reducing future new infections, would thus require intense focus on this population. We would therefore like to recommend specially designed interventions for this sub-typology. It would be important to focus on reduction in number of risky sex acts, enabling periodic HIV testing for HIV uninfected FSW and immediate linkage to care and antiretroviral therapy[17,22,23]. The higher literacy among SB sex workers could be leveraged in developing novel methods of delivery of interventions.

We found a lower HIV prevalence among HB FSWs, though they represent the largest category of FSWs in our study and are largely illiterate. Therefore it is a challenge to ensure that this population maintains low HIV prevalence rates. However due to their home based nature of activity this population poses an important challenge for delivery of prevention interventions[24]. In this study, home based FSWs were older as compared to other types of FSW. We hypothesize that this could be because either they enter sex work at an older age or may be remaining in sex work for longer duration. However our data reveals that, they were 3 times more likely to have been involved in sex work only for shorter period (1–3 years) as compared to BB FSWs. This could support the hypothesis that they enter sex work at later age, probably due to emergent financial needs. We suggest structural interventions like microfinance loans and skill-based education at appropriate times for these women [25–27].

Similar to the other studies conducted among Bar-B FSWs of Mumbai and Navi Mumbai area [25, 26][28,29], we also report that the Bar-B FSWs were younger in age than other FSWs. The probable reason being the client’s demand for young girls who are more valued and solicitation also involves recreational dancing acts. An intervention project conducted in Mumbai and Thane district revealed that regular meetings with bar managers and owners to sensitize them on issues of Bar-B FSWs are extremely important[15]. Since bar managers and owners offer a gateway to approach this population, interventions among new recruits to the bar could be a focused strategy that can yield good prevention benefits.

There are a few limitations to the study. Being a cross sectional study, it was difficult to assign temporality between the HIV status and behavioral characteristics of the respondents [30]. In our study, the data was collected from intervened FSWs attending the TI sites, it may not be completely representative of all the FSW population, especially those who are hidden and hard to reach for the program. However, leads identified in the study can still help the program to focus on the most vulnerable FSWs using tailored strategies. The typologies in the study are considered as mutually exclusive in the TI program, but in real life these may be overlapping in few cases[3,14].

## Conclusion

Findings from this study reveal the heterogeneity in characteristics and vulnerabilities of different typologies of FSWs in Maharashtra. In our study, SB FSWs have emerged as one of the most important typologies with the highest HIV prevalence amongst all non-brothel based FSWs, more frequent sex acts despite lower client loads, more likelihood of being located in a HIV high burden district and vulnerabilities which are similar to BB FSWs.

As India advances towards achievement of 90-90-90 goals with ‘test and treat all’ strategy these findings may help in planning enhanced prevention interventions and develop a focused strategy for SB FSWs. The higher literacy rate in this subpopulation could be an enabler for qualitative research to inform the design of novel interventions for sustaining the decreasing trends in HIV epidemic and moving towards the end game.
